# Giant Pancreatic Acinar Cell Carcinoma: A Case Report and Review of Molecular Pathogenesis and Emerging Therapies

**DOI:** 10.7759/cureus.103628

**Published:** 2026-02-14

**Authors:** Rio Akiyama, Yujo Kawashita, Miku Ochiai, Masaki Tateishi, Takashi Ueda, Masayuki Nakamura, Koya Umeda, Seiko Harada, Sosei Abe, Masashi Haraguchi, Junzo Yamaguchi, Yasuo Washida, Yoichi Hachitanda

**Affiliations:** 1 Surgery, Fukuoka Seishukai Hospital, Fukuoka, JPN; 2 Radiology, Fukuoka Seishukai Hospital, Fukuoka, JPN; 3 Pathology, Fukuoka Seishukai Hospital, Fukuoka, JPN

**Keywords:** distal pancreatectomy(dp), molecular pathogenesis, pancreatic acinar cell carcinoma, pancreatic ductal adenocarcinoma, targeted therapy, β-catenin

## Abstract

Pancreatic acinar cell carcinoma (ACC) is a rare pancreatic malignancy with molecular and clinical features distinct from pancreatic ductal adenocarcinoma (PDAC). We report a 58-year-old man with a 15-cm ACC of the pancreatic body and tail treated by distal pancreatectomy and splenectomy. Complete resection was achieved despite tumor size and surface fissuring suggestive of impending rupture. Operative time was 282 minutes with blood loss of 280 mL. Histopathology showed acinar architecture with B-cell lymphoma 10 (Bcl-10) positivity and nuclear beta-catenin accumulation, consistent with Wnt pathway activation. To clarify the biological differences between ACC and PDAC, we compared their molecular and clinicopathological features. Our analysis demonstrates that ACC's unique molecular profile and growth characteristics enabled successful surgical resection in this giant tumor. This case illustrates how systematic comparison of ACC with PDAC provides insights into the distinct biological behaviors of these pancreatic malignancies. We review current therapeutic advances in PDAC, including targeted therapies and immunotherapy approaches, which may inform future treatment strategies for ACC.

## Introduction

Pancreatic cancer represents an increasingly significant threat to global health. Unlike most malignancies for which mortality rates have declined with therapeutic advances, mortality due to pancreatic cancer continues to rise worldwide, with projections indicating age-standardized mortality rates will exceed 15 per 100,000 population in high-income countries by 2030 [1,2]. This rising trend establishes pancreatic cancer as a formidable challenge for the coming decades.

Pancreatic malignancies comprise multiple histological subtypes with vastly different biological behaviors. Pancreatic ductal adenocarcinoma (PDAC), representing over 90% of cases, is characterized by infiltrative growth, abundant desmoplastic stroma, and dismal prognosis with five-year survival rates of 5%-10% [3]. The extensive desmoplastic reaction and infiltrative pattern often preclude complete surgical resection even in apparently localized disease. In contrast, pancreatic acinar cell carcinoma (ACC), comprising only 1%-2% of pancreatic malignancies, demonstrates distinct molecular pathogenesis and biological behavior [4,5]. ACC arises from the enzyme-producing acinar cells of the exocrine pancreas rather than the ductal epithelial cells that give rise to PDAC, a fundamental difference that contributes to its unique biological characteristics.

We report a case of giant pancreatic ACC measuring 15 cm that, despite massive size with evidence of impending rupture, was successfully treated with complete surgical resection. This case exemplifies how ACC's expansile growth pattern with minimal desmoplasia enables curative surgery even in massive tumors - a stark contrast to PDAC where surgical curability rapidly diminishes with increasing size. Through molecular comparison with PDAC, we elucidate the biological basis for these divergent behaviors and discuss emerging therapeutic strategies that may transform pancreatic cancer management.

## Case presentation

In August 2022, a 58-year-old man presented to the Department of Surgery at Fukuoka Seishukai Hospital, Fukuoka, Japan, with a three-month history of progressive left upper abdominal pain and back pain, accompanied by loss of appetite. His medical history was unremarkable. Social history was significant for heavy alcohol consumption (beer 1,400 mL/day) and smoking (20 cigarettes/day).

Physical examination revealed no evidence of anemia or jaundice. A large, child's head-sized mass with tenderness was palpable in the left upper abdomen. Laboratory investigations demonstrated mildly elevated inflammatory markers (C-reactive protein (CRP) 2.56 mg/dL; reference range <0.30 mg/dL) and elevated gamma-glutamyl transferase (gamma-glutamyl transferase (γ-GTP) 427 U/L; reference range 10-60 U/L), while tumor markers including carbohydrate antigen 19-9 (CA19-9, 18.0 U/mL; reference range <37 U/mL) and carcinoembryonic antigen (CEA, 3.2 ng/mL; reference range <5.0 ng/mL) remained within normal limits (Table 1).

**Table 1:** Laboratory Data on Admission. Abnormal values are indicated in the "Status" column: ↑ indicates values above the reference range; ↓ indicates values below the reference range. Abbreviations: AST, aspartate aminotransferase; ALT, alanine aminotransferase; ALP, alkaline phosphatase; γ-GTP, gamma-glutamyl transferase; BUN, blood urea nitrogen; eGFR, estimated glomerular filtration rate; CRP, C-reactive protein; PT-INR, prothrombin time-international normalized ratio; CA19-9, carbohydrate antigen 19-9; CEA, carcinoembryonic antigen.

Parameter	Value	Unit	Reference Range	Status
Biochemistry				
Total Protein	6.4	g/dL	6.5-8.3	↓
Albumin	3.7	g/dL	3.8-5.3	↓
Total Bilirubin	0.6	mg/dL	0.2-1.2	
AST	27	U/L	10-40	
ALT	22	U/L	5-45	
ALP	275	U/L	100-325	
γ-GTP	427	U/L	10-60	↑
Total Cholesterol	191	mg/dL	120-220	
Triglycerides	115	mg/dL	30-150	
BUN	5.3	mg/dL	8-20	
Creatinine	0.73	mg/dL	0.6-1.2	
eGFR	85.35	mL/min/1.73m²	>60	
Sodium	144	mEq/L	135-145	
Potassium	4.2	mEq/L	3.5-5.0	
Chloride	106	mEq/L	98-108	
Amylase	91	U/L	40-125	
CRP	2.56	mg/dL	<0.30	↑
Hematology				
White Blood Cells	7266	/μL	4000-9000	
Red Blood Cells	406	×10⁴/μL	400-550	
Hemoglobin	13.2	g/dL	13.5-17.5	
Platelets	30.6	×10⁴/μL	15-35	
Coagulation				
Prothrombin Time	95.5	%	70-130	
PT-INR	1.03		0.85-1.15	
Tumor Markers				
CA19-9	18	U/mL	<37	
CEA	3.2	ng/mL	<5.0	

Complete blood count, renal function, and coagulation parameters were unremarkable.

**Figure 1:**
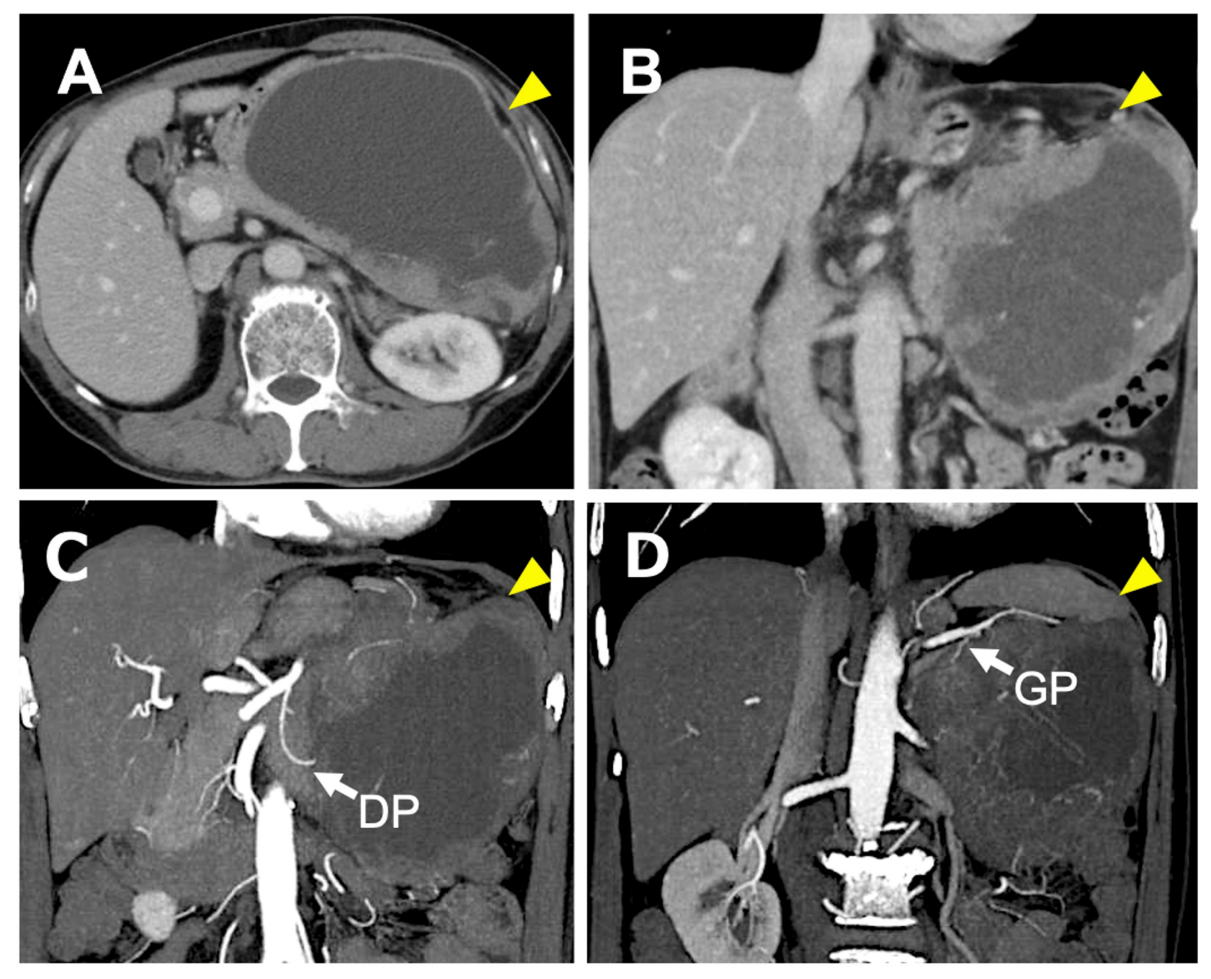
Contrast-enhanced computed tomography (CT) of the abdomen. (A) Axial and (B) coronal images demonstrate a giant heterogeneous tumor measuring approximately 15 cm in maximum diameter, arising in the left upper abdominal cavity. The tumor exhibits internal cystic degeneration consistent with necrosis and prominent peripheral enhancement (arrowheads). (C, D) Computed tomography angiography (CTA) reveals that the dorsal pancreatic artery (DP) and the great pancreatic artery (GP) serve as feeding vessels to the solid component of the tumor (arrows). The tumor shows an expansile growth pattern with relatively well-defined margins and no dilation of the main pancreatic duct, imaging features that help distinguish acinar cell carcinoma (ACC) from pancreatic ductal adenocarcinoma (PDAC).

Contrast-enhanced computed tomography (CT) of the abdomen revealed a massive heterogeneous tumor around the left upper abdominal cavity, measuring approximately 15 cm in maximum diameter (Figure 1).

Although the tumor demonstrated contact with the stomach, left adrenal gland, and spleen, CT angiography (CTA) demonstrated that the dorsal pancreatic artery and the great pancreatic artery served as feeders to the solid component of the lesion, supporting the diagnosis of a pancreatic-origin tumor. Prominent tumor neovascularization with vascular encasement was observed, along with extensive internal cystic degeneration consistent with tumor necrosis. Importantly, the tumor exhibited an expansile growth pattern with relatively well-defined margins and no evidence of main pancreatic duct dilation. These imaging characteristics, combined with normal CA19-9 levels, raised clinical suspicion for ACC rather than PDAC.

Intraoperative exploration confirmed an elastically firm tumor with remarkable neovascularization. Critically, partial fissuring was identified on the tumor surface, indicating impending rupture. Meticulous dissection was performed along the retropancreatic fascia. Due to the massive tumor size, the pancreas was transected first using an Endo GIA stapler (black cartridge, 60 mm; Covidien, Mansfield, MA, USA) directly above the portal vein, followed by splenic artery ligation. The tumor was removed en bloc with the distal pancreas and spleen, achieving grossly complete resection. The operative time was 282 minutes with estimated blood loss of 280 mL without transfusion. The postoperative course was uneventful, with discharge on postoperative day 13. The patient has been followed for 39 months postoperatively (surgery: August 3, 2022; last follow-up: November 2025) with regular physical examination, tumor markers, and contrast-enhanced CT imaging. Postoperative tumor markers at three months showed CA19-9 of 16.2 U/mL and CEA of 2.9 ng/mL, both within normal limits. At 39 months, CA19-9 was 17.8 U/mL and CEA was 3.1 ng/mL, remaining normal throughout the entire follow-up period. Serial imaging demonstrated no evidence of local recurrence or distant metastasis. The patient returned to full daily activities within two months and remains in excellent health with no disease-related symptoms or pancreatic insufficiency.

**Figure 2:**
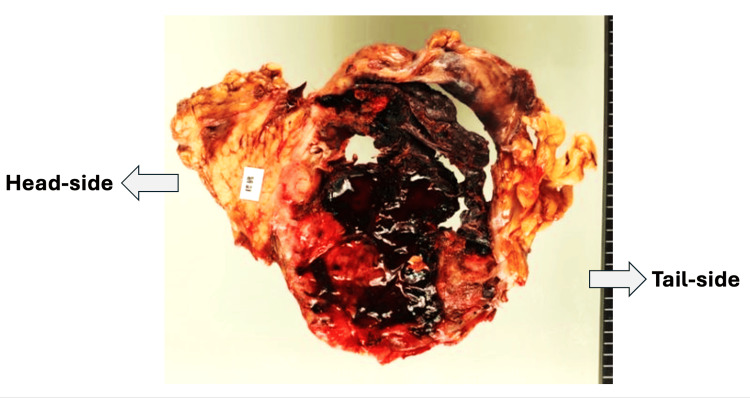
Macroscopic findings of the resected specimen. The tumor exhibits extensive central hemorrhagic necrosis with a relatively well-defined boundary separated by a fibrous capsule-like structure from normal pancreatic tissue, reflecting minimal desmoplasia characteristic of acinar cell carcinoma (ACC).

Macroscopic examination revealed a large, solid tumor with extensive central hemorrhagic necrosis (Figure 2).

The tumor exhibited a relatively well-defined boundary with surrounding pancreatic parenchyma, separated by a fibrous capsule-like structure.

**Figure 3:**
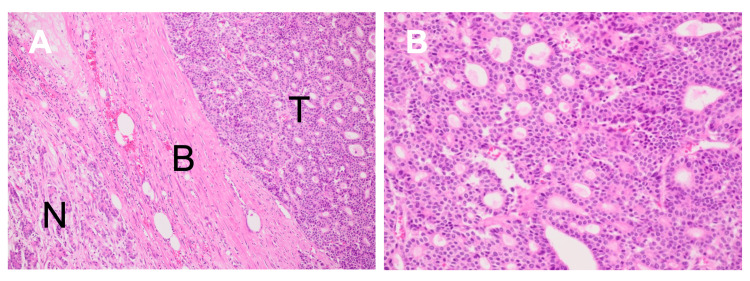
Histopathological findings (hematoxylin and eosin staining). (A) Low-power view demonstrating normal pancreatic parenchyma (N), the tumor boundary with fibrous component (B), and tumor area (T). The fibrous capsule separates the tumor from normal pancreatic tissue, reflecting the minimal desmoplasia characteristic of acinar cell carcinoma (ACC) in contrast to pancreatic ductal adenocarcinoma (PDAC). (B) High-power view showing tumor cells arranged in acinar patterns with granular eosinophilic cytoplasm and round nuclei with prominent nucleoli.

Histopathological examination demonstrated a malignant epithelial neoplasm composed of cells arranged in acinar and trabecular patterns (Figure 3).

The tumor cells exhibited granular eosinophilic cytoplasm and round to oval nuclei with prominent nucleoli, characteristic of acinar differentiation. A fibrous component separated the neoplasm from adjacent normal pancreatic parenchyma. Extensive areas of hemorrhagic necrosis were confirmed histologically.

**Figure 4:**
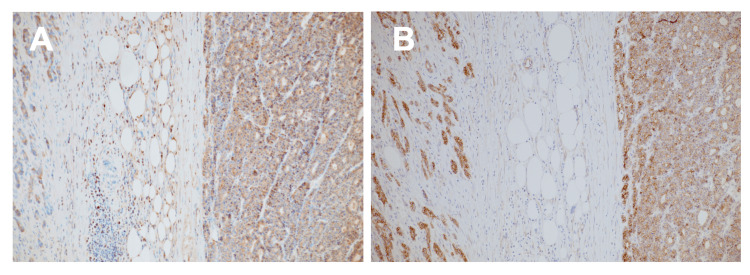
Immunohistochemical findings. (A) B-cell lymphoma 10 (Bcl-10) immunostaining showing strong cytoplasmic positivity in tumor cells, confirming acinar differentiation. (B) Beta-catenin immunostaining demonstrating nuclear accumulation in tumor cells, indicative of aberrant Wnt/beta-catenin pathway activation characteristic of acinar cell carcinoma (ACC) rather than the Kirsten rat sarcoma viral oncogene homolog (KRAS)-driven pathogenesis of pancreatic ductal adenocarcinoma (PDAC).

Immunohistochemical (IHC) analysis revealed strong cytoplasmic positivity for B-cell lymphoma 10 (Bcl-10), confirming acinar differentiation. Additionally, nuclear accumulation of beta-catenin was observed in tumor cells, indicative of aberrant Wnt/beta-catenin pathway activation (Figure 4).

Based on these morphological and immunohistochemical findings, the final diagnosis was pancreatic acinar cell carcinoma.

## Discussion

Pancreatic acinar cell carcinoma represents a clinically significant entity that differs fundamentally from PDAC in molecular pathogenesis, biological behavior, and surgical outcomes. We undertook a systematic comparative analysis with PDAC to clarify these distinctions, as we believe this contrastive approach provides clearer understanding of ACC's unique characteristics and explains the successful surgical management of this giant tumor (Table 2) [3-18].

**Table 2:** Systematic Comparison of Clinicopathological and Molecular Characteristics Between Acinar Cell Carcinoma and Pancreatic Ductal Adenocarcinoma This comparative framework clarifies the biological distinctions that explain divergent surgical outcomes. Abbreviations: ACC, acinar cell carcinoma; PDAC, pancreatic ductal adenocarcinoma; AFP, alpha-fetoprotein; NSE, neuron-specific enolase; CA19-9, carbohydrate antigen 19-9; CEA, carcinoembryonic antigen; APC, adenomatous polyposis coli; BRCA2, breast cancer type 2 susceptibility protein; KRAS, Kirsten rat sarcoma viral oncogene homolog; TP53, tumor protein p53; SMAD4, mothers against decapentaplegic homolog 4; CDKN2A, cyclin-dependent kinase inhibitor 2A; TGF-β, transforming growth factor-beta; IHC, immunohistochemistry; Bcl-10, B-cell lymphoma 10; CK7, cytokeratin 7; CK19, cytokeratin 19; MUC1, mucin 1.

Characteristic	ACC	PDAC
Clinical Features		
Incidence	Rare (1-2%) [4,5]	Most common (~90%) [3]
Predominant site	Body-tail	Head
Tumor markers	AFP, NSE, Lipase (CA19-9 negative)	CA19-9, CEA
Imaging features	Well-defined, expansive, often cystic, no duct dilation	Ill-defined, infiltrative, ductal dilation
5-year survival	20-40% [17,18]	5-10% [3]
Molecular and Pathological Features		
Cell of origin	Acinar cells	Ductal epithelium
Key genetic mutations	APC/β-catenin [8,9], BRCA2 [7,11]	KRAS, TP53, SMAD4, CDKN2A [6]
Growth pattern	Expansile	Infiltrative
Desmoplasia	Low [5]	High (TGF-β/SMAD4 pathway) [13]
Surgical margin	Clear	Unclear
IHC markers	Bcl-10, trypsin, β-catenin (nuclear)	CK7, CK19, MUC1

Additionally, we discuss the emerging therapeutic strategies for PDAC and address the important clinical heterogeneity within ACC that significantly impacts prognosis.

The decision to systematically contrast ACC with PDAC throughout this discussion is deliberate and serves multiple purposes. First, PDAC represents the overwhelming majority (>90%) of pancreatic malignancies, making it the reference point for most clinicians encountering pancreatic tumors. Second, the stark biological differences between ACC and PDAC - in molecular drivers, stromal characteristics, and surgical behavior - are best understood through direct comparison. Third, this contrastive framework helps explain why our 15-cm ACC was surgically resectable whereas a PDAC of similar size would almost certainly be unresectable due to extensive local invasion. Understanding ACC in isolation provides incomplete insight; understanding it in the context of PDAC illuminates the unique biology that enables different therapeutic approaches.

The molecular landscape of ACC is fundamentally distinct from that of PDAC, as comprehensively demonstrated through comparative genomic studies. While PDAC is characterized by near-universal KRAS mutations (>90% of cases) along with frequent alterations in tumor protein p53 (TP53), cyclin-dependent kinase inhibitor 2A (CDKN2A), and mothers against decapentaplegic homolog 4 (SMAD4) [6], these canonical driver mutations are exceedingly rare in ACC. Comprehensive genomic profiling has demonstrated KRAS mutations in fewer than 2% of ACC cases [7].

Regarding tumor markers, while CA19-9 is elevated in the majority of PDAC cases and serves as a useful monitoring tool, it was within normal limits both preoperatively and throughout the 39-month postoperative period in our ACC case (preoperative: 18.0 U/mL; three months: 16.2 U/mL; 39 months: 17.8 U/mL). Similarly, CEA remained consistently normal (preoperative: 3.2 ng/mL; three months: 2.9 ng/mL; 39 months: 3.1 ng/mL). This pattern is consistent with prior reports indicating that CA19-9 is frequently normal in ACC, limiting its utility for diagnosis but potentially serving as a reliable longitudinal marker when baseline values are established. The stability of these markers over more than three years of follow-up provides reassurance regarding the absence of occult recurrence and supports their continued use in surveillance protocols for this patient.

Instead, ACC is characterized by alterations in the Wnt/beta-catenin signaling pathway, present in approximately 20%-25% of cases [8,9]. These include both inactivating mutations in adenomatous polyposis coli (APC) and activating mutations in catenin beta 1 (CTNNB1, the beta-catenin gene). When APC function is lost or beta-catenin harbors stabilizing mutations, beta-catenin accumulates in the cytoplasm, translocates to the nucleus, and activates T-cell factor/lymphoid enhancer-binding factor (TCF/LEF)-dependent transcription of target genes promoting cellular proliferation [10]. In our case, immunohistochemical demonstration of nuclear beta-catenin accumulation supports the presence of aberrant Wnt pathway activation, consistent with ACC molecular pathogenesis rather than the KRAS-driven oncogenesis characteristic of PDAC.

Additionally, mutations in DNA repair genes, particularly breast cancer type 2 susceptibility protein (BRCA2), occur in approximately 20% of ACC cases [7,11]. These findings suggest that subsets of ACC tumors exhibit genomic instability and may potentially respond to targeted therapies such as poly(adenosine diphosphate-ribose) polymerase (PARP) inhibitors through mechanisms of synthetic lethality [12], an approach distinct from PDAC therapeutic strategies.

Perhaps the most clinically consequential biological difference between ACC and PDAC is the degree of desmoplastic stromal reaction. PDAC is characterized by abundant desmoplastic stroma, often comprising up to 80% of tumor volume, mediated largely by transforming growth factor-beta (TGF-β) signaling abnormalities and SMAD4 inactivation [13]. This extensive desmoplasia contributes to the infiltrative growth pattern, unclear surgical margins, vascular encasement, and therapeutic resistance characteristic of PDAC. The dense fibrotic stroma creates a physical barrier to drug delivery and provides a supportive niche for cancer cells, contributing to PDAC's notorious resistance to chemotherapy.

In stark contrast, ACC demonstrates minimal desmoplasia, reflecting its origin from acinar cells and the absence of molecular drivers of stromal reaction seen in PDAC [5]. While precise morphometric quantification was not performed in the current case, histopathological examination revealed stromal content estimated at less than 5% of tumor volume, consistent with the characteristically low desmoplastic reaction of ACC and in marked contrast to the 80% stromal content typical of PDAC [13]. This fundamental difference manifests as the expansile growth pattern, well-circumscribed margins, and relatively clearer surgical planes observed in ACC, as exemplified in our case. The fibrous capsule identified histopathologically at the tumor-pancreas interface represents compression of normal tissue rather than desmoplastic invasion, facilitating complete surgical resection. These characteristics underscore why pursuing R0 (microscopically margin-negative) resection in ACC is more frequently achievable than in PDAC - the tumor biology is conducive to complete excision, unlike the infiltrative nature of PDAC that often involves critical vascular structures even in smaller tumors.

While our case demonstrates the surgical curability of ACC due to its favorable biology, the majority of pancreatic malignancies are PDAC, for which curative therapies remain elusive. We include discussion of emerging PDAC therapies for two important reasons: first, to provide comprehensive context for understanding the spectrum of pancreatic malignancies and the challenges they present; second, because molecular insights from PDAC research may ultimately inform ACC management, particularly for the subset of aggressive or metastatic ACC cases that share some biological features with PDAC.

KRAS mutations, present in over 90% of PDAC cases, have historically been considered undruggable targets. However, the development of KRAS G12C-specific inhibitors represents a paradigm shift. Sotorasib, a small molecule that selectively and irreversibly inhibits KRAS G12C, has shown promising activity in the subset of pancreatic cancer patients (1%-2%) harboring this specific mutation. In phase 1 and 2 trials of 38 heavily pretreated patients with KRAS G12C-mutated advanced pancreatic cancer, sotorasib demonstrated a 21% objective response rate with median progression-free survival of 4.0 months and overall survival of 6.9 months [14]. While modest compared to response rates in KRAS G12C-mutated non-small cell lung cancer, these results represent clinically meaningful activity and provide proof-of-concept that KRAS inhibition is viable. Ongoing research explores inhibitors targeting the more common KRAS G12D mutation, potentially benefiting a broader patient population.

Another revolutionary approach involves personalized messenger ribonucleic acid (mRNA) neoantigen vaccines. Rojas et al. reported groundbreaking results from a phase 1 trial of autogene cevumeran, an individualized mRNA vaccine targeting up to 20 patient-specific neoantigens identified through next-generation sequencing of surgically resected PDAC tumors [15]. Patients received sequential treatment with atezolizumab (anti-programmed death-ligand 1 (PD-L1)), autogene cevumeran, and modified folinic acid, fluorouracil, irinotecan, and oxaliplatin (FOLFIRINOX) chemotherapy. Among 16 treated patients, eight (50%) developed vaccine-induced T-cell responses. At 18-month median follow-up, patients with vaccine-induced responses had significantly longer recurrence-free survival (median not reached) compared to non-responders (13.4 months, P=0.003).

Extended follow-up at 3.2 years revealed even more impressive findings: vaccine-expanded cluster of differentiation 8-positive (CD8+) T-cell clones demonstrated remarkable longevity with an average estimated lifespan of 7.7 years, and responders continued to demonstrate prolonged recurrence-free survival [16]. These results suggest that despite PDAC's relatively low mutation burden, personalized mRNA vaccines can induce durable anti-tumor immunity that correlates with clinical benefit. Ongoing randomized phase 2 studies are comparing autogene cevumeran combined with standard adjuvant chemotherapy versus chemotherapy alone in resected PDAC, potentially establishing a new standard of care that could also be explored in high-risk ACC patients.

While ACC generally demonstrates more favorable prognosis than PDAC, it is crucial to recognize significant heterogeneity within ACC that has important clinical implications. Recent large-scale analyses reveal that 30%-50% of ACC patients present with metastatic disease at diagnosis, with the liver being the most common site (68%), followed by peritoneum (19%) and distant lymph nodes (14%) [17,18]. This high rate of metastatic presentation challenges the traditional view of ACC as an indolent malignancy.

Poorly differentiated ACC demonstrates more aggressive behavior with higher rates of lymphovascular invasion, early metastasis, and worse overall survival [19]. A population-based study analyzing contemporary trends revealed concerning findings: the incidence of ACC is increasing, and the rate of distant metastasis is rising with survival rates worsening over time [19]. This suggests that ACC may require more aggressive treatment and surveillance than previously recognized, and that the biological heterogeneity within ACC encompasses both favorable and aggressive variants.

It is noteworthy that despite the massive tumor size (15 cm) in the current case, pathological examination revealed pN0 status with no lymph node involvement. This finding contrasts with the general observation that 30%-50% of ACC patients present with metastatic disease [17,18] and underscores an important principle: tumor size alone does not predict nodal involvement or metastatic potential in ACC. Rather, biological behavior is more strongly influenced by histological grade, differentiation status, and molecular characteristics than by anatomical extent. The expansile rather than infiltrative growth pattern - a direct consequence of minimal desmoplasia - enabled complete resection despite dimensions (15 cm) that would virtually guarantee unresectability in PDAC due to inevitable vascular involvement and regional invasion. The 39-month disease-free interval further validates that achieving R0 resection in large but well-circumscribed ACC can result in outcomes comparable to smaller tumors, provided favorable histological features are present.

For patients with resected localized disease, median survival ranges from 36 to 47 months, compared to only 14 months for those with unresectable or metastatic disease [20]. The overall five-year survival for all stages combined ranges from 10% to 40%, reflecting this substantial heterogeneity [17,18]. Importantly, ACC variants with specific histological features - such as poorly differentiated morphology, high mitotic index, or mixed acinar-neuroendocrine components - are associated with worse prognosis and more aggressive clinical behavior. Recognition of these high-risk features is essential for appropriate treatment planning and patient counseling.

For metastatic ACC, platinum-based regimens (folinic acid, fluorouracil, and oxaliplatin (FOLFOX) or FOLFIRINOX) have shown superior outcomes compared to gemcitabine-based protocols [18], suggesting shared chemosensitivity with colorectal and pancreatic adenocarcinomas. This observation underscores the importance of comprehensive molecular profiling to identify actionable targets such as BRCA1/2 mutations (present in approximately 20% of ACC), which may benefit from PARP inhibitors [11], or microsatellite instability, which may respond to immune checkpoint inhibitors. The heterogeneity within ACC demands individualized treatment approaches based on both histological features and molecular characteristics.

Complete surgical resection with negative margins (R0 resection) remains the cornerstone of treatment for localized ACC. The biological characteristics of ACC - expansile growth, well-defined margins, and minimal desmoplasia - often facilitate complete resection compared to PDAC [4,5]. In our case, despite the massive tumor size of 15 cm and evidence of impending rupture with surface fissuring, the distinct tumor margins enabled R0 resection without the need for extended vascular or multivisceral resection that would likely be required for a PDAC of comparable size. The importance of R0 resection cannot be overstated, as it represents the only potentially curative treatment and significantly impacts long-term survival, particularly for patients with favorable histological features.

Pathological examination confirmed the final staging as pT3N0M0 (Stage IIB according to the American Joint Committee on Cancer (AJCC), 8th edition), with the tumor measuring 15 cm and extending beyond the pancreas into peripancreatic soft tissue but without lymph node involvement (0/12 lymph nodes examined) or distant metastasis. While Stage IIB ACC carries a guarded prognosis with reported median survival of 36-47 months in contemporary series [20], several favorable prognostic factors were present in this case: achievement of R0 resection with microscopically negative margins (closest margin: 8 mm), absence of lymph node metastasis despite examination of an adequate number of nodes, well-to-moderately differentiated histology without poorly differentiated or anaplastic components, and absence of lymphovascular or perineural invasion.

The patient's disease-free status at 39 months postoperatively now exceeds the median survival reported for resected ACC in most series and approaches the point at which long-term survival probability increases substantially. Combined with the favorable pathological features noted above, this outcome suggests the patient may be among the 20%-40% of ACC patients who achieve five-year survival [17,18]. The extended follow-up period without evidence of recurrence provides increasingly strong evidence of potential cure, though continued vigilant surveillance remains essential given the documented heterogeneity within ACC, with some patients experiencing late recurrence beyond three years.

This study has several limitations inherent to a single case report. The generalizability of findings is limited, as individual cases may not reflect the full spectrum of ACC behavior. Comprehensive molecular profiling including next-generation sequencing for KRAS, TP53, SMAD4, and CDKN2A mutations - the canonical driver mutations of PDAC - was not performed. Such testing would have definitively confirmed the absence of these PDAC-associated alterations and provided detailed characterization of ACC-specific genetic alterations. While immunohistochemical demonstration of nuclear β-catenin accumulation confirms aberrant Wnt pathway activation characteristic of ACC rather than KRAS-driven pathogenesis, genetic sequencing of APC and CTNNB1 would have provided definitive molecular confirmation. Additionally, testing for BRCA1/2 mutations (present in approximately 20% of ACC cases [7,11]) or microsatellite instability status was not performed; identification of such alterations might have expanded therapeutic options including PARP inhibitors or immune checkpoint inhibitors, respectively. These molecular data would have been particularly valuable given the patient's excellent long-term outcome, as they might help identify predictive biomarkers for favorable prognosis in ACC. The absence of preoperative tissue diagnosis through endoscopic ultrasound-guided fine-needle aspiration (EUS-FNA), while consistent with our institutional practice to avoid potential tumor seeding, limited our ability to establish definitive preoperative diagnosis and conduct molecular analysis on preoperative specimens.

## Conclusions

Pancreatic ACC is a biologically distinct entity from PDAC, characterized by unique molecular alterations, including aberrations in the APC/beta-catenin signaling pathway rather than KRAS mutations, and minimal desmoplastic stromal reaction. Through systematic comparative analysis, we demonstrate that these molecular features contribute to an expansile growth pattern and relatively well-defined surgical margins that facilitate complete resection even in massive tumors - a stark contrast to the infiltrative, desmoplasia-rich PDAC where surgical curability diminishes rapidly with increasing size.

However, ACC demonstrates substantial clinical heterogeneity, with 30%-50% of patients presenting with metastatic disease and poorly differentiated variants exhibiting aggressive behavior comparable to PDAC. Recognition of this heterogeneity is essential for appropriate treatment planning and patient counseling. While recent advances in KRAS-targeted therapy and personalized mRNA neoantigen vaccines offer hope for transforming PDAC outcomes, ACC patients may also benefit from molecular profiling to identify actionable targets. Surgical resection aiming for R0 margins should remain the primary treatment strategy for localized disease when feasible, as the unique biology of ACC enables curative resection in cases that would be deemed unresectable if they were PDAC. Comprehensive genomic profiling may expand therapeutic options across the spectrum of pancreatic malignancies, ultimately improving outcomes for both common and rare histological subtypes.
